# Altered Topological Properties of Brain Networks in Social Anxiety Disorder: A Resting-state Functional MRI Study

**DOI:** 10.1038/srep43089

**Published:** 2017-03-07

**Authors:** Hongru Zhu, Changjian Qiu, Yajing Meng, Minlan Yuan, Yan Zhang, Zhengjia Ren, Yuchen Li, Xiaoqi Huang, Qiyong Gong, Su Lui, Wei Zhang

**Affiliations:** 1 Mental Health Center, West China Hospital, Sichuan University, Chengdu 610041, China; 2 State Key Laboratory of Biotherapy, West China Hospital, Sichuan University, Chengdu 610041, Sichuan, China; 3 Huaxi MR Research Center (HMRRC), Department of Radiology, West China Hospital, Sichuan University, Chengdu 610041, China; 4 Radiology Department of the Second Affiliated Hospital, Wenzhou Medical University, Wenzhou, Zhejiang 325027 China

## Abstract

Recent studies involving connectome analysis including graph theory have yielded potential biomarkers for mental disorders. In this study, we aimed to investigate the differences of resting-state network between patients with social anxiety disorder (SAD) and healthy controls (HCs), as well as to distinguish between individual subjects using topological properties. In total, 42 SAD patients and the same number of HCs underwent resting functional MRI, and the topological organization of the whole-brain functional network was calculated using graph theory. Compared with the controls, the patients showed a decrease in 49 positive connections. In the topological analysis, the patients showed an increase in the area under the curve (AUC) of the global shortest path length of the network (*L*_*p*_) and a decrease in the AUC of the global clustering coefficient of the network (*C*_*p*_). Furthermore, the AUCs of *L*_*p*_ and *C*_*p*_ were used to effectively discriminate the individual SAD patients from the HCs with high accuracy. This study revealed that the neural networks of the SAD patients showed changes in topological characteristics, and these changes were prominent not only in both groups but also at the individual level. This study provides a new perspective for the identification of patients with SAD.

Social anxiety disorder (SAD) is the most common anxiety disorder, and it is characterized by fear and avoidance of social situations associated with being observed or evaluated by others or a fear of embarrassing oneself^1^. Recent evidence has suggested that abnormal cerebral functioning is involved in the pathogenesis of SAD. Neuroimaging studies have found an increase in the regional activity of the limbic and paralimbic regions including the amygdala and anterior cingulate, as well as reduced activity in the striatal and parietal areas both in groups^2^ and at the individual level^3^. For example, using a linear support vector machine (SVM), we identified regional homogeneity (ReHo) in the default mode network, dorsal attention network, self-referential network, and sensory networks, thus distinguishing the patients with SAD from the healthy controls with a diagnostic accuracy of 76.25%^3^.

However, the findings from the regional analysis were inconsistent and distributed across the entire cerebral region of the patients with SAD, which may be due to the effects of medication or sample heterogeneity. These inconsistent findings can also be attributed to subtle and widespread white matter deficits across the brain, which are not perfectly integrated by traditional regional or voxel-based analyses. Therefore, connectomics has been used to evaluate the whole brain as an interconnected network^4^.

Graph theory provides powerful mathematical tools to study the behaviour of complex systems of interacting elements^5^. It has been widely used to characterize local and distributed interactions in the brain^6^, and altered topological characteristics in functional brain networks have been observed in psychiatric disorders such as schizophrenia^7^,^8^, major depressive disorder^9^,^10^, and obsessive-compulsive disorder^11^. In particular, we found significant correlations between functional connectivity and disease severity in specific regions of resting-state networks (RSNs), including the medial and lateral prefrontal cortex, as well as the parietal and occipital regions, in SAD patients using independent component analysis^12^.

Previous studies of SAD have indicated that the human connectome can yield potential biomarkers for SAD. However, few studies have explored the topology of the functional network in SAD. Last year, we used functional connectivity strength (FCS) to examine the cortical hubs in SAD using the graph theory method and found that the patients with SAD had disrupted cortical hubs during resting states, a discovery that provides novel insights into the pathophysiological mechanisms of SAD^13^. In addition to cortical hubs, “small-world” parameters and network efficiency, which can indicate the organization of the global network, are also important in graph theory analysis. However, no studies have explored both the global and regional topological properties of SAD. Although there are many topological indexes in the graph theory method, it is unclear whether the topological characteristics are altered and whether these indexes can be used to identify SAD patients individually.

The present study analysed the network topology of the brain in patients with SAD using intrinsic functional networks. The topological organization of the functional brain network based on resting-state functional connectivity and the linkage between the characteristics of the brain networks and the patients’ clinical conditions were assessed in SAD patients, as well as healthy controls (HCs). We hypothesized that the global and regional topological properties could be used to distinguish SAD patients from HCs by multivariate pattern analysis (MVPA).

## Materials and Methods

### Subjects

We initially recruited 47 SAD patients and 45 healthy controls, all Han Chinese, for this study. Data from five patients and three controls were discarded because of excessive head movement during the MRI scan. Therefore, 42 SAD patients (26 males and 16 females with a mean age of 27.33 ± 7.159 years) and 42 controls (26 males and 16 females with a mean age of 29.83 ± 8.748 years) were finally included in the statistical analysis. The psychiatric diagnostic classification of the participants was based on the Structured Clinical Interview for DSM-IV Axis I Disorders (SCID)^14^ conducted by two attending psychiatrists and a trained interviewer.

All of the subjects in the SAD group were in accordance with the DSM-IV criteria for SAD. Four of the patients had been taking a stable dosage of a selective serotonin reuptake inhibitor for at least 4 weeks (two patients were treated with paroxetine (20 mg/day), one patient was treated with paroxetine (10 mg/day), and one patient was treated with paroxetine (20 mg/day), as well as tandospirone (20 mg/day)). However, some of the patients had discontinued their use of psychotropic medications due to poor responses at least 2 weeks prior to the baseline MRI scan. We also recruited 45 controls who had never been diagnosed with SAD or any other psychiatric disorders.

The exclusion criteria included any history of neurological disease, bipolar disorder, alcohol and/or other substance abuse/dependence, major head injury involving loss of consciousness for more than 10 minutes, other DSM-IV axis I diagnoses, and mental retardation, as well as subjects with metal implants (e.g., surgical clips or pacemakers). According to the Structured Clinical Interview for DSM-IV^14^, the participants in the SAD group met the criteria for the following current comorbid diagnoses: major depression (N = 2) and generalized anxiety disorder (N = 1).

All of the participants were assessed using the Liebowitz Social Anxiety Scale Self-Report (LSAS), the Hamilton Rating Scale for Depression (HAMD-24), and the Hamilton Rating Scale for Anxiety (HAMA-14), and all of the patients provided informed consent. This study was approved by the Medical Ethics Committee of West China Hospital, Sichuan University, and all of the experiments were performed in accordance with the Declaration of Helsinki.

### Image acquisition

We used a 3.0 T magnetic resonance scanner (Siemens 3.0 T Trio Tim, Germany) with a 12-channel phase array head coil. Each subject was positioned supine in the MRI scanner with foam padding to reduce head movements. The fMRI blood-oxygen-level-dependent (BOLD) images were acquired by a gradient-echo-planar imaging (EPI) sequence (TR/TE = 2,000/30 ms; flip angle = 90°). The slice thickness was 5 mm with a matrix size of 64 × 64, resulting in voxels of 3.75 × 3.75 × 5 mm^3^. The subjects were instructed to close their eyes, let their minds wander, and relax, but not to fall asleep during the scan, which lasted 6.8 min (205 volume), as described in our previous study^15^.

### Data preprocessing

Functional image preprocessing and statistical analyses were conducted using the Data Processing Assistant for Resting-State fMRI (DPARSF_V4.0) in DPABI (http://rfmri.org/dpabi)^16^, which is based on Statistical Parametric Mapping (SPM8, http://www.fil.ion.ucl.ac.uk/spm) and the Matlab toolbox (http://www.brain-connectivity-toolbox.net). The DPARSF includes powerful and updated preprocessing steps and has been used in hundreds of research projects^17^. The first five time points were discarded for scanner calibration and acclimation of the subjects to the scanning conditions. Thus, 200 time points from the rest condition time series were used for network analysis. For each participant, the images were corrected for differences in the intravolume acquisition time between slices using sinc interpolation and then corrected for intervolume geometric displacement due to head movement using a six-parameter (rigid-body) spatial transformation^10^. Data from five patients and three healthy controls were discarded because their heads moved more than 3 mm of translation or 3 degrees of rotation in any direction. After these corrections, the realigned images were spatially normalized to the EPI template in SPM8, and each voxel was resampled to 3 × 3 × 3 mm^3^ without spatial filtering. For network metrics calculation, the images were smoothed using a 4-mm full-width half-maximum (FWHM) isotropic Gaussian filter^18^. For each run, the nuisance terms were regressed from the resting-state BOLD time series through multiple linear regressions. These nuisance regressors included the following: i) linear and quadratic trends, ii) a Friston 24-parameter autoregressive model that included current and past position parameters and adequately addressed head motion effects^18^, iii) white matter and CSF signals, which were masked based on SPM apriori, and iv) white matter and cerebrospinal fluid time series. The summary scalars of both the gross (maximum and root mean square) and micro (mean frame-wise displacement) head movements were matched between the two groups (all p > 0.15). Finally, the corrected BOLD time series were low-pass filtered using a cut-off frequency of 0.01–0.1 Hz to reduce the low frequency drift and high frequency noise.

### Network construction

#### Node definition

A network is composed of nodes and edges between nodes. In the brain, the nodes represent the brain regions, and the edges represent the statistical relationships of BOLD signals across different regions. Since the atlas of Automated Anatomical Labeling^19^ (AAL) has been most commonly used in previous studies and is widely accepted in neuroimaging studies, we used the AAL, which includes 90 areas in the whole brain that represent 90 nodes of the brain network. The names and indexes of the 90 areas (45 for each hemisphere) are listed in Table S1 in the Supplementary Material.

#### Edge definition

To define the network edges, the interregional resting-state functional connectivity (RSFC) was calculated using Pearson correlations between the regional mean time series of all possible pairs of brain regions. The correlation coefficients were transformed to z-scores via Fisher’s transformation to improve normality^20^. We constructed a 90 × 90 correlation matrix for each subject.

### Network analysis

#### Threshold Selection

Based on the 90 × 90 correlation matrix for each subject, we constructed a binary undirected graph, and a sparsity threshold was used to measure all of the correlation matrices. Due to the difficulty involved in selecting a single threshold, empirical studies^9^,^21^ were used with a wide range of 0.10 ≤ sparsity ≤ 0.34 (interval = 0.01), in which the “small-world” parameters could be properly estimated^22^ and the number of spurious edges was minimized^23^,^24^,^25^. According to the previous study, there were two criteria considered when the threshold was generated: 1) the average degree (see Supplementary Material for the definition) over all of the nodes of each thresholded network was larger than 2 × log(N), with N = 90 denoting the number of nodes; and 2) the small-worldness (see Supplementary Material for definition) of the thresholded networks was larger than 1.1 for all participants^9^. To make sure all/most of the nodes were connected for each subject after thresholding, we also used the largest component size (that is, the number of nodes in the largest connected component divided by the number of all of the available network nodes N^26^).

**Figure 1:**
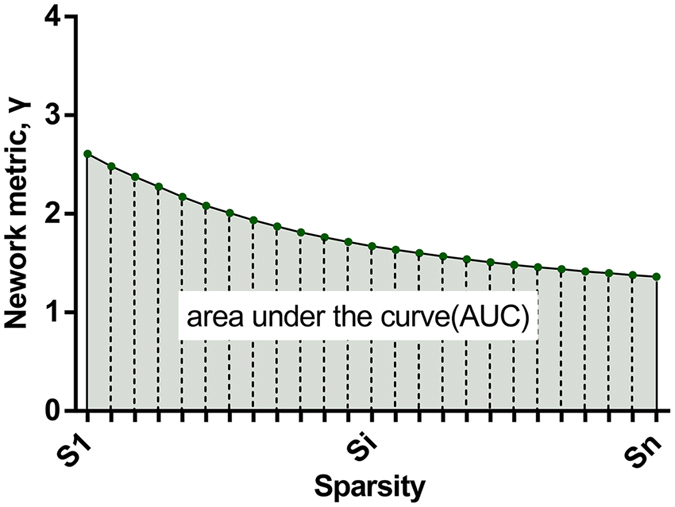
Graph showing area under the curve (AUC) for a network. The shaded area represents AUC. Metric Y was calculated over the sparsity threshold range of S1 to Sn at an interval. In the current study, S1 = 0.10, Sn = 0.34, and the interval = 0.01.

Due to the ambiguous biological explanation for negative correlations^27^,^28^, we restricted our analyses to positive correlations. We calculated both the global and node network metrics at each sparsity, and the area under the curve (AUC) for each network metric, which provides a summarized scalar for the topological characterization of brain networks (Fig. 1), was calculated.

#### Network Metrics

We analysed the global metrics in this study using the following parameters: (1) The “small-world” parameter clustering coefficients included the shortest path length, normalized characteristic path length (λ), normalized clustering coefficient (γ), and small-worldness (σ). The shortest path length is defined as the shortest mean distance from a particular vertex to all other vertices^22^,^29^. Thus, a smaller path length represents greater integration. The clustering coefficient is defined as the fraction of a vertex’s neighbours that are neighbours themselves^29^, while a larger clustering coefficient represents greater segregation^30^. The path length and clustering coefficient were normalized by the related mean metrics of the 100 random networks. These random networks had the same number of nodes, edges, and degree distributions as the real brain networks. (2) Network efficiency included the local efficiency of the whole network (E_loc_), the global efficiency of the network (E_glob_), the nodal global efficiency of the node (nodalE_glob_), and the nodal local efficiency of the node (nodalE_loc_). (3) Nodal centrality (the degree number of nodes (nodalDeg)) was the final parameter. These definitions and descriptions of the metrics are listed in Supplementary Material and are provided in a reference 29.

All of the network metrics were calculated using the GRaph thEoreTical Network Analysis (GRETNA) toolbox (https://www.nitrc.org/projects/gretna/)^31^, and this method of network construction and calculation has been used in previous studies of brain networks^21^,^32^,^33^. The brain networks were visualized using the BrainNet Viewer^34^.

### Statistical analysis

#### Functional network connectivity analysis between the SAD patients and the HCs

We performed two-sample, two-tailed t-tests on all 7560 of the possible connections represented in the 90 × 90 correlation matrices between the patients and the controls^35^ using GRETNA, and the false discovery rate (FDR) procedure was applied to correct for multiple comparisons.

#### Group comparisons based on topological metrics

We used non-parametric permutation tests (10,000 permutations) adopting the Matlab language to test the intragroup differences in the brain network metrics, and gender and age were treated as the unconcerned covariates for comparisons. Furthermore, the problem of multiple comparisons was addressed by testing the graph-based metrics for survival using a Benjamini-Hochberg false discovery rate correction at the expected significance level of 0.05^36^. Spearman’s correlation coefficient was used to calculate the associations between the network metrics and the clinical variables in the SAD groups.

#### Multivariate pattern analysis

We used a multivariate pattern analysis (MVPA) to explore the role of these functional connectivity and network parameters in distinguishing the SAD patients from the healthy controls. To reduce the data dimensions and improve the performance of the classifier^37^,^38^, we used feature selection based on two-sample, two-tailed t-tests. Thus, the functional connectivity (FC) and network metrics with significant differences between groups were treated as discriminant features (p < 0.05, uncorrected). Maximum uncertainty linear discriminate analysis (MLDA) employing a maximum entropy covariance selection method instead of the within-class scatter matrix was used as the classifier^39^,^40^,^41^. The performance of the classifier was estimated using a leave-one-out cross-validation (LOOCV) approach, and these steps were supported by multi-modal imaging and multi-level characterization with multi-classifier (M3), which has been made publicly available at: http://www.nitrc.org/projects/pare/ 39. Finally, we used a permutation test to infer the significance of the classifier performance by random disruption of the label in all of the SAD patients and healthy controls, and the class validation procedure was repeated 1000 times. Thus, we obtained a distribution of the classifier performance with random labels, which was used to calculate the z-score and p value.

## Results

### Demographic data and clinical comparisons

There were no significant differences between the SAD group and the control group in terms of age, sex, and, education (p > 0.05; Table 1).

### Disrupted Functional Network Connectivity in SAD

**Figure 2:**
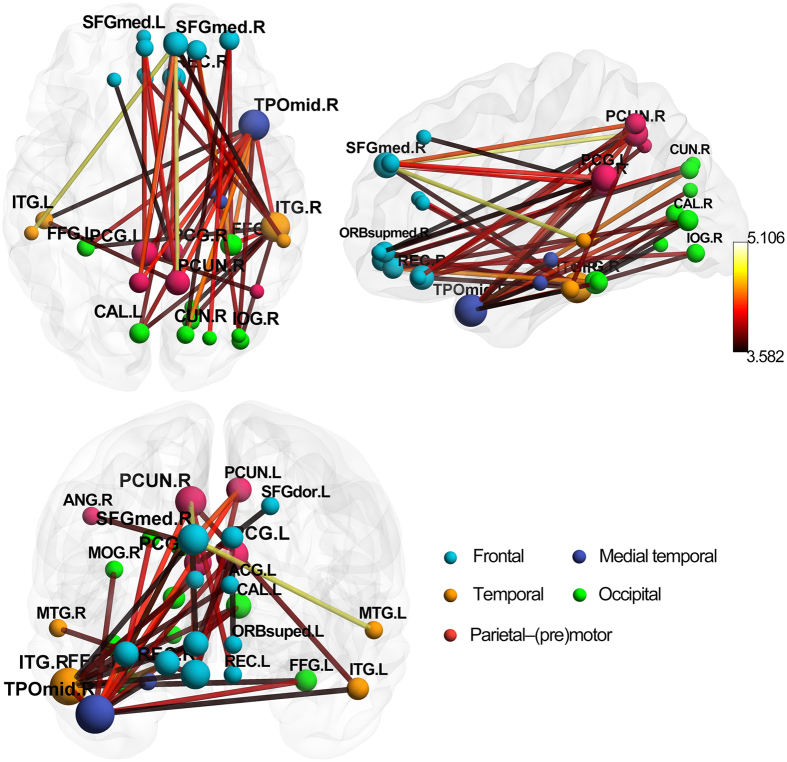
Decreased functional connections in the SAD group compared with the control group. There are 49 decreased connections which were significantly (FDR corrected p = 0.05) abnormal in the patients. All of 32 brain regions are marked by using different colored spheres (different colors represent distinct brain classification) and further mapped onto the cortical surfaces at the lateral, medial and top views, respectively, by using the BrainNet Viewer package (www.nitrc.org/projects/bnv). The size of the spheres represents the number of altered connections they involved. The color of the edges represents the t value of the comparisons, and the color bar is on the left side. **ACG**, anterior cingulate and paracingulate gyri; **ANG**, angular gyrus; **CAL**, calcarine fissure and surrounding cortex; **CUN**, cuneus; **FFG**, fusiform gyrus; **IOG**, inferior occipital gyrus; **ITG**, inferior temporal gyrus; **MOG**, middle occipital gyrus; **MTG**, middle temporal gyrus; **ORBsuped**, superior frontal gyrus, medial orbital; **PCG**, posterior cingulate gyrus; **PCUN**, precuneus; **REC**, gyrus rectus; **SFGdor**, superior frontal gyrus, dorsolateral; **SFGmed**, superior frontal gyrus, medial; **TPOmid**, temporal pole: middle temporal gyrus.

Compared with the HCs, 49 connections showed a significant decrease in positive connections in the SAD patients (p < 0.05, FDR-corrected). The most altered connections involved the frontal, occipital, parietal–(pre)motor, and temporal regions, and all of the altered connection pairs are listed in Fig. 2 and Supplement Table S2. Additionally, we used a stricter significance level of p < 0.01 (FDR-corrected) and found two decreased connections in the SAD patients, including the right superior frontal gyrus, medial–left posterior cingulate gyrus and the right superior frontal gyrus, medial–right precuneus. These results reveal that the abnormal correlations in the SAD patients are universal and more related to the default mode network.

### Global parameters of brain networks

**Figure 3:**
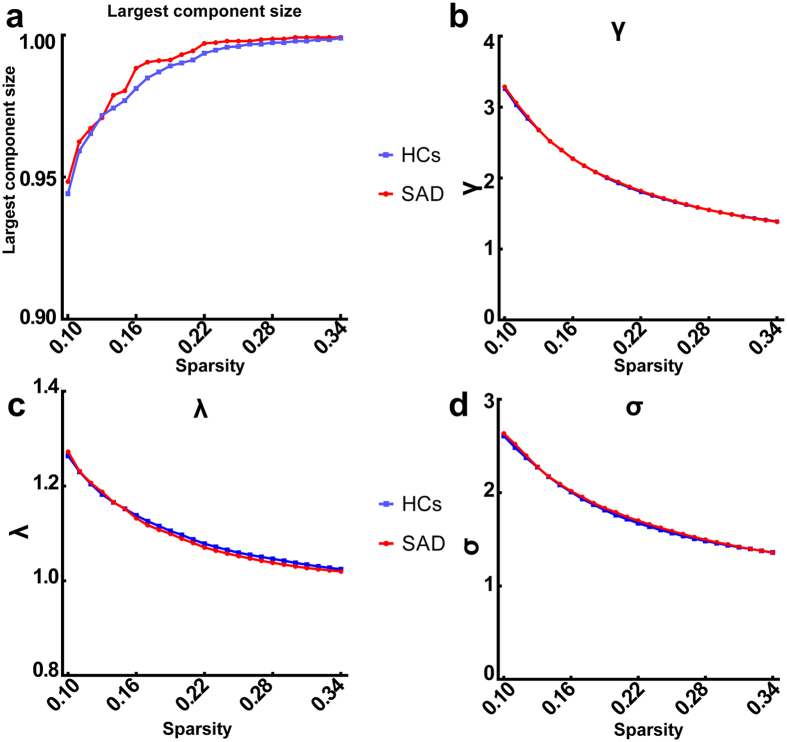
Largest component size and “Small-world” parameters in the defined threshold range. Graphs show that in the defined threshold range, both the SAD and control groups exhibited (**a**) the number of nodes in the largest connected component divided by all the available nodes N, (**b**) the normalized clustering coefficient, (**c**) the normalized characteristic path length, and (**d**) small-world measure.

To make sure that all/most of the nodes were connected after the strongest thresholding, we examined the largest component size at all thresholds (see Fig. 3a) and found that most of the nodes (0.944) were connected even at the strongest threshold (sparsity = 0.10).

**Figure 4:**
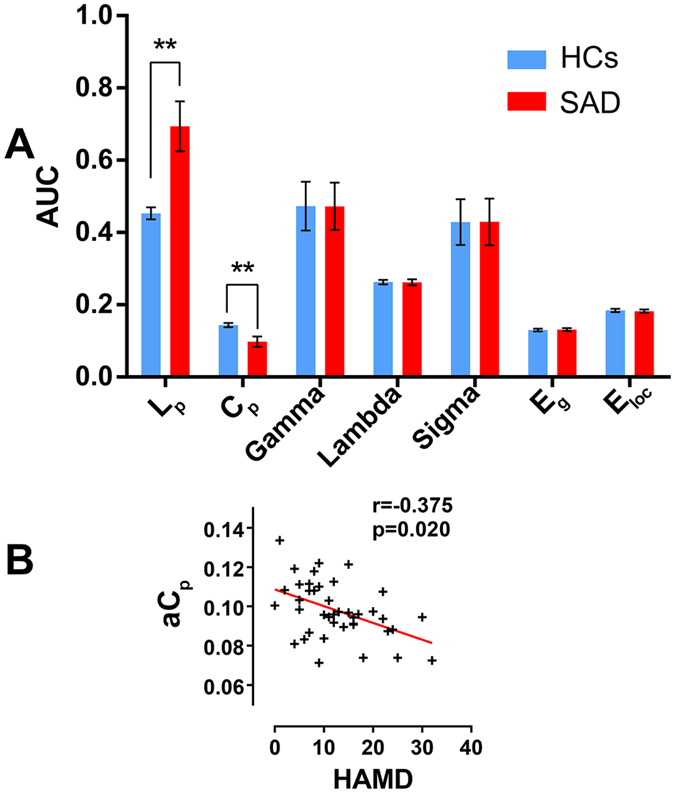
Global topology of functional connectivity networks in the SAD and control groups. Part A: Topological properties of functional connectivity networks in SAD and controls are shown in different colors. The error bars represent standard deviation. ** Group difference is significant at the 0.01 level (FDR corrected) based on non-parametric permutation tests. Part B: Scatter plots in part B show significant correlation between the global topological characteristics and HAMD scores in the SAD group (p < 0.05). **SAD**, social anxiety disorder; **HCs**, health controls; **AUC**, area under the curve; **L**_**p**_, shortest path length; **C**_**p**_, clustering coefficient; **γ**, the normalized clustering coefficient; **λ**, the normalized characteristic path length; **σ**, small-world measure; **E**_**glob**_, global efficiency; **E**_**loc**,_ local efficiency; **aL**_**p**_ AUC of shortest path length; **aC**_**p**_, AUC of clustering coefficient; HAMD, Hamilton Rating Scale for Depression.

There were no significant differences between the SAD group and control group in local and global efficiencies, λ, γ, and σ (Fig. 4A). In both groups, the values of λ hovered at approximately 1, γ significantly exceeded 1, and σ exceeded 1, suggesting that both the patients with SAD and the controls showed “small-world” organization in resting states (Fig. 3b–d).

Compared with the HC group, an increased AUC of the shortest path length of the network (*L*_*p*_) and a decreased clustering coefficient of the network (*C*_*p*_) were observed in the patients with SAD (p < 0.01). A negative correlation was also found between the AUC of *C*_*p*_ (a*C*_*p*_) and the HAMD scores in the SAD group (r = 0.361, p = 0.026). (Fig. 4B). Nevertheless, the correlation was no longer significant after Bonferroni correction.

### Regional parameters of brain networks

After FDR correction, no significant differences in the AUC of nodal degree (anodalDeg) and the AUC of nodal global efficiency (anodalE_glob_) were observed between the SAD and control groups.

**Figure 5:**
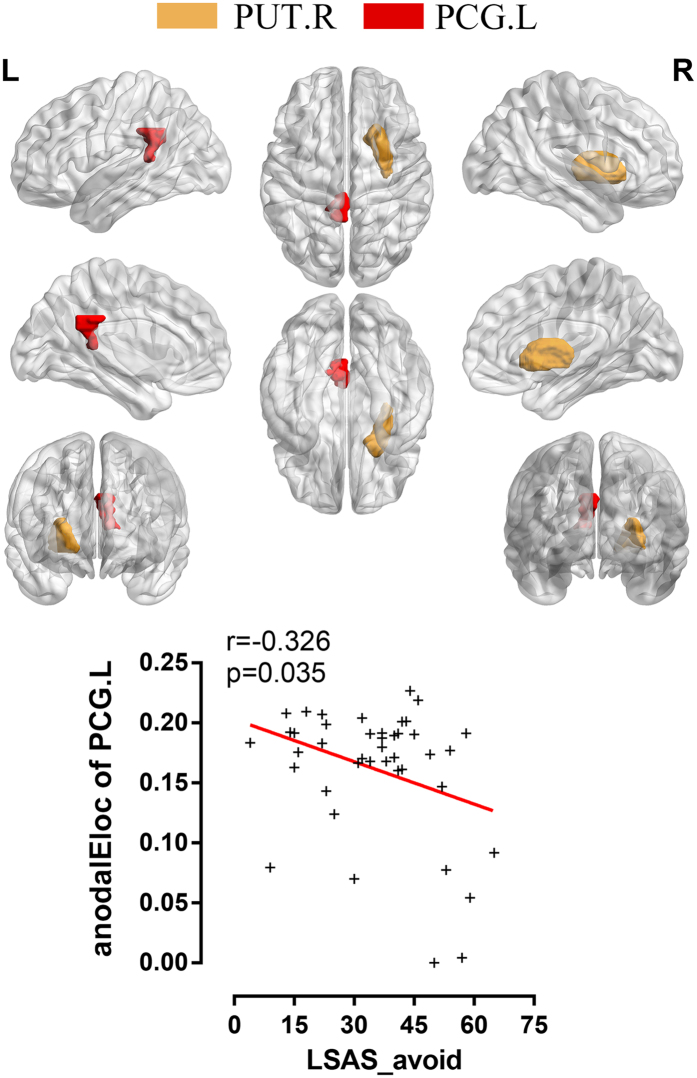
Altered regional brain topological parameters in the SAD. The colored areas show the location of left posterior cingulate gyrus and the right putamen in the Automated Anatomical Labeling atlas. Scatter plots show significant correlation between the nodal local efficiency in left posterior cingulate gyrus and avoidance score of LSAS in the SAD group (p < 0.05). **PUT.R**, right putamen; **PCG.L**, left posterior cingulate gyrus; **anodalE**_**loc**_, area under the curve of nodal local efficiency; **LSAS_avoid**, avoidance score of Liebowitz Social Anxiety Scale.

Regarding local efficiency, a decreased AUC of nodal local efficiency (anodalE_loc_) was observed in the left posterior cingulate gyrus (PCG) of the SAD patients compared with the controls, and the nodal local efficiency in the right putamen (PUT) was also increased (p < 0.05, FDR corrected) (see Fig. 5). The anodalE_loc_ of the left PCG and the avoidance score on the LSAS showed a significant negative correlation in the SAD patients (r = −0.326, p = 0.035), but the correlation was no longer significant after Bonferroni correction.

### Discriminant analysis

**Figure 6:**
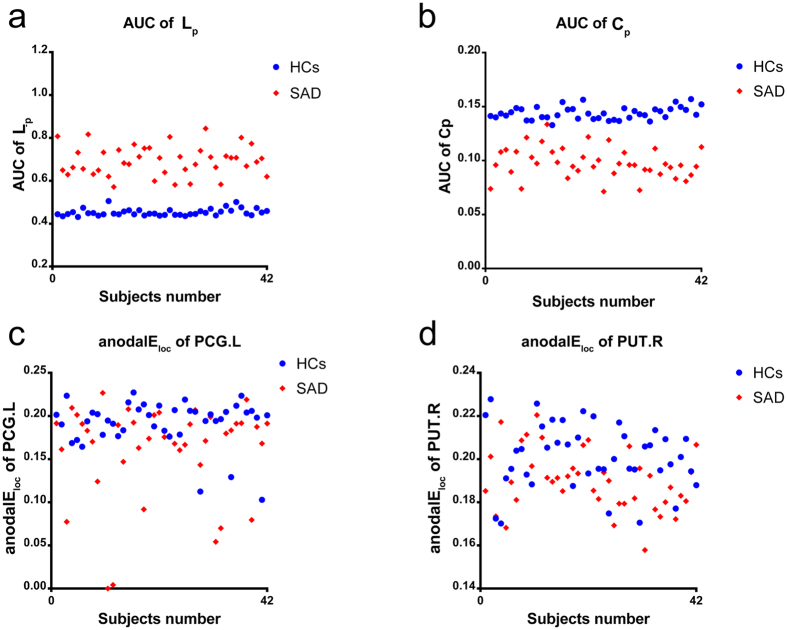
The individual network features in both SAD and control groups. The network features which can discriminate SAD from healthy controls effectively were abstracted. (**a**) the AUC of *L*_*p*_, (**b**) the AUC of *C*_*p*_, (**c**) the anodalE_loc_ of PCG.L and (**d**) the anodalE_loc_ of PUT.R. **SAD**, social anxiety disorder; **HCs**, health controls; **AUC**, area under the curve; **L**_**p**_, shortest path length; **C**_**p**_, clustering coefficient; **anodalE**_**loc**_, area under the curve of nodal local efficiency; **PCG.L**, left posterior cingulate gyrus; **PUT.R**, right putamen.

Only the altered FC and network metrics including the 49 decreased FCs, the AUCs of *L*_*p*_ and *C*_*p*_, and the anodalE_loc_ in the left PCG and right PUT were investigated to separately distinguish the SAD patients from the HCs. Because of the stricter inclusion criteria of the features before M3, all of the features we chose were retained after the looser feature selection of M3. These network metrics were abstracted individually and are shown in Fig. 6.

Based on the 49 altered FC analysis findings, we found that the discrimination ability (accuracy = 0.667, sensitivity = 0.738, specificity = 0.595) was not significant after the nonparametric permutation test (z = 1.728). We also calculated the feature weights of all of the features, which represent the contribution to the classification^39^. The feature weights of all of the pairs in the M3 analysis are listed in Supplement Table S2, and the feature weights of the topological parameters are listed in the Table 2.

We found that the AUC of *L*_*p*_ and the AUC of *C*_*p*_ effectively discriminated between the two groups separately (a*L*_*p*_: accuracy = 0.988, sensitivity = 0.976, specificity = 1; a*C*_*p*_: accuracy = 0.964, sensitivity = 0.929, specificity = 1), which was significantly above the random level (a*L*_*p*_:z = 12.554; a*C*_*p*_: z = 12.783). Only a single patient was miscategorised as a control among the 84 subjects. Combining the AUC of *L*_*p*_ and the AUC of *C*_*p*_ yielded an accuracy rate that was similar to the AUC of *L*_*p*_ alone (accuracy = 0.988, sensitivity = 0.976, specificity = 1, z = 12.514). The classification performance and feature weights of the topological parameters are listed in the Table 2.

We used the anodalE_loc_ in the left PCG and right PUT to distinguish the SAD patients from the HCs, and we also found that the local nodal efficiency of the left PCG and right PUT in the resting-state network significantly distinguished the two groups (accuracy = 0. 714, sensitivity = 0. 738, specificity = 0.691, z = 5.237).

## Discussion

The present study used resting-state fMRI to explore changes in the brain topology of SAD patients. To our knowledge, this is the first study to investigate the “small-world” brain network in SAD patients. Compared with controls, the SAD patients had 49 decreased connections, which involved the frontal, occipital, parietal–(pre)motor, and temporal regions. In the global topological analysis, an increased AUC of *L*_*p*_ and a decreased AUC of *C*_*p*_were found in the patients with SAD. In the local regions, an increased anodalE_loc_ of the right PUT was observed in the patients with SAD, while the anodalE_loc_ of the left PCG was reduced. These findings reveal a shifting of the “small-world” properties in SAD patients during resting states and the critical role of the PUT and PCG in the pathogenesis of SAD. These altered topological metrics may be caused by a disruption of functional connectivity in SAD. Furthermore, these differences were used to effectively discriminate between the individual patients with SAD and the HCs with an accuracy of 98.8%.

In the functional network connectivity analysis, the major altered connections involved the frontal, occipital, parietal–(pre)motor, and temporal regions. Compared with the HCs, all of the changed connections were decreased, which may indicate that network efficiency is disrupted in patients with SAD. The attenuation of the FC between the frontal and occipital lobes was consistent with the results of our previous study of SAD, which had a small sample size^42^. The frontal, occipital, parietal–(pre)motor, and temporal regions are related to the functions of cognition, emotion, and memory, and these regions were examined in previous functional MRI studies of SAD^43^. Moreover, the areas in the default mode network (DMN)^44^,^45^,^46^ including the precuneus, posterior cingulate gyrus, angular gyrus, middle temporal gyrus, medial frontal gyrus, and superior frontal gyrus were obviously involved. The DMN is thought to be involved in episodic memory^47^, self-projection^48^, and social cognition^49^. Impairment of the DMN network in SAD might be relevant to the development of feelings of wariness concerning the judgement of others and may be related to the self-focused attention^49^. The cuneus and calcarine fissure in the occipital lobe are involved in converging facial expressions^50^,^51^ and contextual self-descriptions^52^. Therefore, the decreased correlations between the median prefrontal cortex and occipital lobe might indicate that SAD is related to abnormal processing of the nonverbal information relayed by human facial expressions^42^.

According to the currently fashionable idea, the brain network can be divided into the regular network, the random network, and the “small-world” network. The regular network is characterized by a higher clustering coefficient and a longer shortest path length. The random network has a lower clustering coefficient and a shorter shortest path length. The “small-world” network has a higher clustering coefficient similar to the regular network and a shortest path length that is similar to the random network^22^. In the current study, we found that both the patients with SAD and the controls had a λ ≈ 1, a γ ≫ 1, and a σ that was >1, indicating the presence of “small-world” brain networks in both groups. The functional connection was correlated with the structural connections in the brain, and “small-world” functional and structural networks occur in the human brain, as well as at the cellular level in other animals^5^. Numerous studies using electroencephalography (EEG)^53^, magnetoencephalography (MEG)^54^, and fMRI^55^ have reported “small-world” properties of whole-brain functional networks in humans. In the “small-world” brain network, functional integration and segregation are the two major organizational principles. An optimal brain requires a balance between global integration and local specialization of brain functional activity^56^ and achieves a maximal communication speed with minimal energy consumption^57^. Among these topological indices, *C*_*p*_ and *L*_*p*_ represent segregation and integration, respectively. *C*_*p*_ is equivalent to the fraction of nodal neighbours that are also neighbours to each other^22^, which reflects the local efficiency or fault tolerance of a network^58^. Thus, a decrease in *C*_*p*_ indicates local efficiency or fault tolerance, which decreases functional segregation when disrupted in the brains of patients with SAD. *L*_*p*_ is a global feature that indicates the information-carrying capacity of the brain. A lower *L*_*p*_ value ensures global information transmission capacity and provides a reliable base of functional integration in the brain^59^,^60^. The increase in the AUC of *L*_*p*_ in our study suggests a disruption of global information transmission in the brains of patients with SAD. Although the brain networks of the SAD patients and controls showed “small-world” characteristics, the increased AUC of *C*_*p*_ and decreased AUC of *L*_*p*_ represent disruption in the brain networks in terms of functional integration and separation in the SAD patients, which may reflect an imbalance of global integration and local specialization. This imbalance suggested a disruption in the energy cost of spontaneous brain activity and was associated with the severity of depression in SAD patients, which was compatible with major depressive disorder^61^.

Among these exploratory correlation analyses with clinical measurements, we found that the AUC of *C*_*p*_ was significantly associated with depression but not social (or general) anxiety. Since patients with depression also suffer from an imbalance of global integration and local specialization^61^, it is not clear if this abnormality is more a function of subclinical depressive symptoms rather than the chosen diagnosis. As the correlation with clinical measures involved exploratory analyses, which did not show significance after multiple comparison corrections, the reproducibility of the correlation between the AUC of *C*_*p*_ and HAMD needs to be verified in future studies. Furthermore, it is common for patients with SAD to suffer from more depressive symptoms. Thus, it was hard to disengage anxiety from depression in this study due to the small sample size. Even though the recruitment of SAD patients with few or no depressive symptoms may solve this problem, the application of the results would be restricted in the real world.

In terms of the regional parameters of the brain networks, the SAD group showed alterations of the anodalE_loc_ in the left posterior cingulate gyrus (PCG) and right putamen (PUT) compared with the HCs, and the reduction in the anodalEloc of the left PCG was negatively associated with the avoidance score of the LSAS in the exploratory analysis. The nodalE_loc_ is a summarized scalar reflecting the transmission efficiency of the network at the local level, and the posterior cingulate has been associated with self-referential functions^62^,^63^, self-focused attention (awareness of self-referent information)^64^, evaluation of self-emotional states^65^, and social behaviour^66^. Neuroimaging studies have suggested that the PCG is attenuated during task conditions^49^,^64^. Furthermore, functional imaging has revealed a reduction in rCBF in the PCG of SAD patients during resting states^67^, and a resting-state fMRI study that used functional connectivity suggested that attenuated functional connectivity in the amygdala and the PCG/precuneus is correlated with higher anxiety^68^. The altered nodal efficiency of the left PCG in our study demonstrated low efficiency of local information transfer and processing around the left PCG, which might be related to avoidance behaviour in SAD patients. The putamen is regarded as one of the sectors of the striatum that is the “emotion guarder”, and it is an important terminal for receiving sensory and emotional information from the prefrontal areas^69^. Striatal dysfunction in SAD patients has been observed in previous neuroimaging studies, and it may be linked to the information processing biases found in SAD patients, including negative interpretation of social events, detection of and obsession with negative responses from other people, and selective recall of negative aspects of social interactions^70^,^71^. The higher AUC of the local nodal efficiency of the PUT in the SAD patients reflects a reduction in regional integration of information in the right PUT, which may represent an information processing bias in SAD patients. Although the power of the nodal local efficiency in the left PCG and right PUT was lower than the AUC of *L*_*p*_ and *C*_*p*_, it indicates an important role for these two brain areas in SAD, especially in avoidance symptoms.

In this study, we used discriminant analysis to distinguish SAD patients from controls, and we found that SAD patients were accurately identified using the AUC of *L*_*p*_ and the AUC of *C*_*p*_. The remarkable accuracy of the discriminant analysis may be attributed to the huge disparity in the AUCs of *L*_*p*_ and *C*_*p*_ between the two groups and at the individual level. Thus, these attributes may facilitate diagnosis, suggesting the need for additional studies.

The study limitations were as follows. First, only the leave-one-out cross-validation (LOOCV) approach was supported in the M3 code. Even though this method has been widely used in research^39^,^72^,^73^,^74^, it is sub-optimal and has been shown to result in inaccurate estimates of the prediction error^75^. It is more appropriate to apply n-fold validation when running the primary analyses on a portion of the patients and healthy controls and then apply the primary results to classify the rest of the subjects. Because the sample size in this study was relatively small and because of the huge disparity in the AUCs of *L*_*p*_ and *C*_*p*_ between the two groups and at the individual level (see Fig. 6), we believe that these results should be independently verified in more samples. Second, compared with other studies, we only explored the network based on the AAL brain atlas, which was the most commonly used in previous studies. Although this is widely accepted in neuroimaging studies, new brain atlases have frequently been used in graph theory, for example, the Power 264-region atlas^72^,^76^, the Harvard-Oxford Structural atlas^77^, and the Dosenbach’s 160 functional atlas^78^,^79^. Future studies should verify our results using these atlases in the analyses of different brain networks. Third, several patients with comorbid conditions were included in the current study. Although SAD was their main diagnosis, comorbid conditions such as depression and generalized anxiety may have affected the results. Therefore, separate analyses of SAD patients with and without comorbid conditions are desirable.

## Conclusion

In summary, using graph theory, the current study found that both the global and regional topological characteristics of the neural networks of SAD patients were less effective. This is the first study to investigate the “small-world” properties of SAD, and the changes were prominent at the group and individual levels, providing a new perspective for distinguishing patients from healthy individuals. However, future studies that focus on these topological attributes and brain areas in different samples are needed.

## Additional Information

**How to cite this article:** Zhu, H. *et al*. Altered Topological Properties of Brain Networks in Social Anxiety Disorder: A Resting-state Functional MRI Study. *Sci. Rep.* **7**, 43089; doi: 10.1038/srep43089 (2017).

**Publisher's note:** Springer Nature remains neutral with regard to jurisdictional claims in published maps and institutional affiliations.

## Supplementary Material

Supplemental Materials

## Figures and Tables

**Table 1:** Demographic information and psychological variables in SAD and control groups.

	SAD	Control	T	P
Age	27.33 ± 7.16	29.79 ± 8.78	1.40	0.16
Female to male ratio	16:26	16:26	—	—
Educational years	12.57 ± 4.27	13.52 ± 14.04	1.05	0.30
LSAS (Mean ± SD)	69.40 ± 26.84	20.29 ± 15.71	10.24	<0.001
Fear of LSAS (Mean ± SD)	34.12 ± 13.00	9.79 ± 7.85	10.38	<0.001
Avoiding of LSAS (Mean ± SD)	35.29 ± 15.05	10.50 ± 9.52	9.02	<0.001
HAMD (Mean ± SD)	12.60 ± 7.63	2.17 ± 2.57	8.40	<0.001
HAMA (Mean ± SD)	14.14 ± 7.67	1.79 ± 2.08	10.08	<0.001
Course of disease	8.86 ± 6.78	—	—	—

**SAD**, social anxiety disorder; **LSAS,** the Liebowitz Social Anxiety Scale Self-Report; **HAMD**, the Hamilton Rating Scale for Depression; **HAMA**, the Hamilton Rating Scale for Anxiety; **CGI-S**, Clinical global impressions severity.

**Table 2:** Classification performance of the single topological parameters and multi-level combinations.

Number of features in classifier	Features	Weight	Accuracy	Sensitivity	Specificity	z
1	aLp	1	0.988	0.976	1	12.554
1	aCp	1	0.964	0.929	1	12.783
2	aLp	0.478	0.988	0.976	1	12.514
2	aCp	0.988				
2	anodalEloc in left PCG	0.228	0. 714	0. 738	0.691	5.237
2	anodalEloc in right PUT	0.719				

**PCG.L**, left posterior cingulate gyrus; **PUT.R**, right putamen.
