# Supplementary material for: Altered Topological Properties of Brain Networks in Social Anxiety Disorder: A Resting-state Functional MRI Study
Source: Sci Rep. 2017 Mar 7;7:43089. doi: 10.1038/srep43089 (PMC5339829; doi:10.1038/srep43089)
Supplement: Supplemental Materials [file srep43089-s1.pdf]

Altered Topological Properties of Brain Networks in Social Anxiety Disorder: A Resting-state Functional MRI Study

Hongru Zhu, M.D., Ph.D., Changjian Qiu, M.D., Ph.D., Yajing Meng, M.D., Ph.D., Minlan Yuan, M.D., Yan Zhang, M.D., Zhengjia Ren, M.D., Ph.D., Yuchen Li, M.D., Xiaoqi Huang, M.D., Ph.D., Qiyong Gong, M.D., Ph.D., Su Lui, M.D., Ph.D., Wei Zhang, M.D.

Supplemental Text

Network Analysis

Small-world analysis.

The small-world parameters of a network (clustering coefficient C_p, and characteristic path length L_p) were originally proposed by Watts and Strogatz [1]. Briefly, the C_p of a network was the average of the clustering coefficients over all nodes, where the clustering coefficient C_i of a node i was defined as the ratio of the number of existing connections among the node's neighbors and all their possible connections. C_p quantified the local interconnectivity of a network. L_p of a network was the shortest path length (number of edges) required to transfer from one node to another averaged over all pairs of nodes. L_p was an indicator of the overall routing efficiency of a network. In our study, we calculated the L_p as the "harmonic mean" distance between all possible pairs of regions to deal with the disconnected graphs dilemma [2]. To estimate the small-world properties, we scaled C_p and L_p derived from the brain networks

with the mean C_{p-s} and L_{p-s} of 100 random networks (i.e., $\gamma = C_p / C_{p-s}$ and $\lambda = L_p / L_{p-s}$) that preserved the same number of nodes, edges and degree distributions as real brain networks. Typically, a small-world network should fulfill the conditions of $\gamma > 1$ and $\lambda \approx 1$ [1], and therefore, the small-worldness scalar $\sigma = \gamma / \lambda$ will be more than 1 [2]. Of note, in the present study the "harmonic mean" distance was employed to calculate the characteristic path length to deal with the possible disconnected graphs dilemma [3].

Network efficiency.

Efficiency is a more biologically relevant metric to describe brain networks from the perspective of parallel information flow that can deal with either the disconnected or nonsparse graphs or both [4]. Network efficiency measures how efficiently information is exchanged over the network. For a network G with N nodes and K edges, the global efficiency (E_{glob}) of G can be computed as [5,6]:

$$E_{glob}(G) = \frac{1}{N(N-1)} \sum_{i \neq j \in G} \frac{1}{d_{ij}}, \quad (1)$$

where d_{ij} is the shortest path length between node i and node j in G. Global efficiency measures the ability of parallel information transmission over the network. The local efficiency of (E_{loc}) G is measured as [5,6]:

$$E_{loc}(G) = \frac{1}{N} \sum_{i \in G} E_{glob}(G_i), \quad (2)$$

where $E_{glob}(G_i)$ is the global efficiency of G_i, the subgraph composed of the neighbors of node i. Local efficiency measures the fault tolerance of the

network, indicating the capability of information exchange for each subgraph when the index node is eliminated.

The nodal local efficiency [4] ($nodalE_{loc}$) of node i is defined in the subgraph of the direct neighbors of i:

$$nodalE_{loc}(G, i) = \frac{1}{N_{Gi}(N_{Gi} - 1)} \sum_{j \neq k \notin Gi} \frac{1}{d_{jk}}, \quad (3)$$

where N_{Gi} is the number of nodes in the subgraph Gi consisting of all of the neighbors of i; and d_{jk} is the length of the shortest path, in terms of tractography-based measures of physical distances, between nodes j and k.

The nodal global efficiency [4] ($nodalE_{glob}$) of node i is computed as:

$$nodalE_{glob}(G, i) = \frac{1}{(N - 1)} \sum_{j \in G, i \neq j} \frac{1}{d_{ij}}, \quad (4)$$

where N is the number of nodes in the network graph G; and d_{ij} is the length of the shortest path, in terms of tractography-based measures of physical distances, between nodes i and j.

Nodal centrality. Degree k_i was used to measure the centrality of a node. It is defined as the number of links connected to the node, and it is a simple measurement of connectivity of a node with the rest of nodes in a network. Hub regions often interact with many other regions in the network and thus have high centrality. Formally, in a network G with N nodes and K edges, the degree k_i (nodalDeg) of node i is defined as [5]:

$$k_i = \sum_{j \in G} a_{ij}, \quad (5)$$

where a_{ij} is the i th row and j th column element of the adjacency matrix.

Though these nodal centrality measurements are correlated each other, they reflect the importance of a node in a network from different aspects as defined above [7].

References

1. Watts DJ, Strogatz SH. Collective dynamics of 'small-world' networks. Nature. 1998. 393(6684): 440-2.
2. Humphries, M. D., Gurney, K. & Prescott, T. J. The brainstem reticular formation is a small-world, not scale-free, network. *Proc. Biol. Sci.* **273**, 503-511 (2006).
3. Newman, M. E. Mixing patterns in networks. *Phys Rev E Stat Nonlin Soft Matter Phys* **67**, 026126 (2003).
4. Latora, V. & Marchiori, M. Efficient behavior of small-world networks. *Phys. Rev. Lett.* **87**, 198701 (2001).
5. Liu, Y. *et al.* Disrupted small-world networks in schizophrenia. *Brain* **131**, 945-961 (2008).
6. Achard, S. & Bullmore, E. Efficiency and cost of economical brain functional networks. *PLoS Comput. Biol.* **3**, e17 (2007).
7. Bullmore, E. & Sporns, O. The economy of brain network organization. *Nat. Rev. Neurosci.* **13**, 336-349 (2012).

Supplement Tables

Table S1. Regions of interest (ROIs) included in AAL-atlas

Index	Regions	Abbr.	Index	Regions	Abbr.
(1,2)	Precental gyrus	PreCG	(47,48)	Lingual gyrus	LING
(3,4)	Superior frontal gyrus, dorsolateral	SFGdor	(49,50)	Superior occipital gyrus	SOG
(5,6)	Superior frontal gyrus, orbital part	ORBsup	(51,52)	Middle occipital gyrus	MOG
(7,8)	Middle frontal gyrus	MFG	(53,54)	Inferior occipital gyrus	IOG
(9, 10)	Middle frontal gyrus, orbital part	ORBmid	(55,56)	Fusiform gyrus	FFG
(11,12)	Inferior frontal gyrus, opercular part	IFGoperc	(57,58)	Postcentral gyrus	PoCG
(13,14)	Inferior frontal gyrus, triangular part	IFGtriang	(59,60)	Superior parietal gyrus	SPG
(15,16)	Inferior frontal gyrus, orbital part	ORBinf	(61,62)	Inferior parietal, but supramarginal and angular gyri	IPL
(17,18)	Rolandic operculum	ROL	(63,64)	Supramarginal gyrus	SMG

(19,20)	Supplementary motor area	SMA	(65,66)	Angular gyrus	ANG
(21,22)	Olfactory cortex	OLF	(67,68)	Precuneus	PCUN
(23,24)	Superior frontal gyrus, medial	SFGmed	(69,70)	Paracentral lobule	PCL
(25,26)	Superior frontal gyrus, medial orbital	ORBsupmed	(71,72)	Caudate nucleus	CAU
(27,28)	Gyrus rectus	REC	(73,74)	Lenticular nucleus, putamen	PUT
(29,30)	Insula	INS	(75,76)	Lenticular nucleus, pallidum	PAL
(31,32)	Anterior cingulate and paracingulate gyri	ACG	(77,78)	Thalamus	THA
(33,34)	Median cingulate and paracingulate gyri	DCG	(79,80)	Heschl gyrus	HES
(35,36)	Posterior cingulate gyrus	PCG	(81,82)	Superior temporal gyrus	STG
(37,38)	Hippocampus	HIP	(83,84)	Temporal pole: superior temporal gyrus	TPOsup
(39,40)	Parahippocampal gyrus	PHG	(85,86)	Middle temporal gyrus	MTG
(41,42)	Amygdala	AMYG	(87,88)	Temporal pole: middle temporal gyrus	TPOmid
(43,44)	Calcarine fissure and surrounding cortex	CAL	(89,90)	Inferior temporal gyrus	ITG
(45,46)	Cuneus	CUN			

The regions are listed in terms of a prior AAL atlas [6]. Odd and even numbers represent brain regions of left and right hemispheres, respectively.

Table S2. Decreased positive functional connectivity in SAD compared with healthy controls

Region 1	Classification	Region 2	Classification	t	weight
Left superior frontal gyrus, dorsolateral	Frontal	Right posterior cingulate gyrus	Parietal–(pre) motor	3.61	0.434
Right superior frontal gyrus, orbital part	Frontal	Left calcarine fissure and surrounding cortex	Occipital	3.80	0.097
Right superior frontal gyrus, orbital part	Frontal	Right inferior occipital gyrus	Occipital	3.93	0.650
Right superior frontal gyrus, orbital part	Frontal	Right fusiform gyrus	Occipital	4.42	0.645

Right middle frontal gyrus, orbital part	Frontal	Right cuneus	Occipital	3.66	0.150
Right middle frontal gyrus, orbital part	Frontal	Right superior occipital gyrus	Parietal–(pre) motor	4.07	0.322
Right middle frontal gyrus, orbital part	Frontal	Right fusiform gyrus	Occipital	3.95	0.204
Left superior frontal gyrus, medial	Frontal	Left posterior cingulate gyrus	Parietal–(pre) motor	4.16	0.060
Left superior frontal gyrus, medial	Frontal	Right posterior cingulate gyrus	Parietal–(pre) motor	4.17	0.313
Right superior frontal gyrus, medial	Frontal	Left posterior cingulate gyrus	Parietal–(pre) motor	5.11	0.260
Right superior frontal gyrus, medial	Frontal	Right posterior cingulate gyrus	Parietal–(pre) motor	4.08	0.041
Right superior frontal gyrus, medial	Frontal	Left precuneus	Parietal–(pre) motor	4.29	0.080
Right superior frontal gyrus, medial	Frontal	Right precuneus	Parietal–(pre) motor	4.89	0.558
Right superior frontal gyrus, medial	Frontal	Left middle temporal gyrus	Temporal	4.83	0.299
Right superior frontal gyrus, medial	Frontal	Right inferior temporal gyrus	Temporal	3.65	0.149
Left superior frontal gyrus, medial orbital	Frontal	Left posterior cingulate gyrus	Parietal–(pre) motor	3.60	0.378
Right superior frontal gyrus, medial orbital	Frontal	Left posterior cingulate gyrus	Parietal–(pre) motor	3.68	0.218
Right superior frontal gyrus, medial orbital	Frontal	Right precuneus	Parietal–(pre) motor	3.59	0.048
Right superior frontal gyrus, medial orbital	Frontal	Right inferior temporal gyrus	Temporal	3.70	0.180
Left gyrus rectus	Frontal	Right precuneus	Parietal–(pre) motor	3.97	0.280
Right gyrus rectus	Frontal	Left posterior cingulate gyrus	Parietal–(pre) motor	3.84	0.113
Right gyrus rectus	Frontal	Left precuneus	Parietal–(pre) motor	3.89	0.086
Right gyrus rectus	Frontal	Right precuneus	Parietal–(pre) motor	4.24	0.443
Right gyrus rectus	Frontal	Right middle temporal gyrus	Temporal	3.86	0.218
Right gyrus rectus	Frontal	Right inferior temporal gyrus	Temporal	3.66	0.151
Left anterior cingulate and paracingulate gyri	Frontal	Right inferior temporal gyrus	Temporal	3.84	0.045

Right anterior cingulate and paracingulate gyri	Frontal	Right inferior temporal gyrus	Temporal	4.06	0.267
Left posterior cingulate gyrus	Parietal–(pre) motor	Right angular gyrus	Parietal–(pre) motor	3.72	0.275
Left posterior cingulate gyrus	Parietal–(pre) motor	Right temporal pole: middle temporal gyrus	Medial temporal	3.84	0.032
Right posterior cingulate gyrus	Parietal–(pre) motor	Right temporal pole: middle temporal gyrus	Medial temporal	3.72	0.183
Right hippocampus	Medial temporal	Right temporal pole: middle temporal gyrus	Medial temporal	3.67	0.529
Right parahippocampal gyrus	Medial temporal	Right temporal pole: middle temporal gyrus	Medial temporal	3.60	0.241
Left calcarine fissure and surrounding cortex	Occipital	Right temporal pole: middle temporal gyrus	Medial temporal	3.88	0.033
Left calcarine fissure and surrounding cortex	Occipital	Right inferior temporal gyrus	Temporal	3.70	0.087
Right calcarine fissure and surrounding cortex	Occipital	Right temporal pole: middle temporal gyrus	Medial temporal	4.24	0.416
Right calcarine fissure and surrounding cortex	Occipital	Right inferior temporal gyrus	Temporal	3.73	0.318
Right cuneus	Occipital	Right temporal pole: middle temporal gyrus	Medial temporal	4.41	0.196
Right lingual gyrus	Occipital	Right inferior temporal gyrus	Temporal	3.58	0.216
Right middle occipital gyrus	Occipital	Right temporal pole: middle temporal gyrus	Medial temporal	3.79	0.159
Right inferior occipital gyrus	Occipital	Right inferior temporal gyrus	Temporal	3.71	0.103
Left fusiform gyrus	Occipital	Right temporal pole: middle temporal gyrus	Medial temporal	3.94	0.141
Left fusiform gyrus	Occipital	Right inferior temporal gyrus	Temporal	3.62	0.206
Right fusiform gyrus	Occipital	Right temporal pole: middle	Medial temporal	4.35	0.199

		temporal gyrus			
Right fusiform gyrus	Occipital	Right inferior temporal gyrus	Temporal	4.12	0.123
Left precuneus	Parietal–(pre) motor	Right temporal pole: middle temporal gyrus	Medial temporal	4.18	0.488
Right precuneus	Parietal–(pre) motor	Right temporal pole: middle temporal gyrus	Medial temporal	4.19	0.394
Right precuneus	Parietal–(pre) motor	Left inferior temporal gyrus	Temporal	3.81	0.320
Right temporal pole: middle temporal gyrus	Medial temporal	Left inferior temporal gyrus	Temporal	3.60	0.193
Right temporal pole: middle temporal gyrus	Medial temporal	Right inferior temporal gyrus	Temporal	4.08	0.051
